# Absolute risk representation in cardiovascular disease prevention: comprehension and preferences of health care consumers and general practitioners involved in a focus group study

**DOI:** 10.1186/1471-2458-10-108

**Published:** 2010-03-04

**Authors:** Sophie Hill, Janet Spink, Dominique Cadilhac, Adrian Edwards, Caroline Kaufman, Sophie Rogers, Rebecca Ryan, Andrew Tonkin

**Affiliations:** 1 Centre for Health Communication and Participation, Australian Institute for Primary Care, La Trobe University, Bundoora, Victoria, 3086, Australia; 2 Public Health Division, National Stroke Research Institute, Level 1 Neurosciences Building, 300 Waterdale Road, Heidelberg Heights, Victoria, 3081, Australia; 3 Department of Primary Care & Public Health, School of Medicine, Cardiff University, 2nd floor Neuadd Meirionnydd, Heath Park, Cardiff, Wales, CF14 4XN, UK; 4 University of Texas Southwestern, Austin, 1313 Red River, Ste 303B, Austin, Texas 78701, USA; 5 Centre for Eye Research Australia, The University of Melbourne, East Melbourne, Victoria, 3002, Australia; 6 Cardiovascular Research Unit, Department of Epidemiology and Preventive Medicine, Monash University, Victoria 3800, Australia

## Abstract

**Background:**

Communicating risk is part of primary prevention of coronary heart disease and stroke, collectively referred to as cardiovascular disease (CVD). In Australia, health organisations have promoted an absolute risk approach, thereby raising the question of suitable standardised formats for risk communication.

**Methods:**

Sixteen formats of risk representation were prepared including statements, icons, graphical formats, alone or in combination, and with variable use of colours. All presented the same risk, i.e., the absolute risk for a 55 year old woman, 16% risk of CVD in five years. Preferences for a five or ten-year timeframe were explored. Australian GPs and consumers were recruited for participation in focus groups, with the data analysed thematically and preferred formats tallied.

**Results:**

Three focus groups with health consumers and three with GPs were held, involving 19 consumers and 18 GPs.

Consumers and GPs had similar views on which formats were more easily comprehended and which conveyed 16% risk as a high risk. A simple summation of preferences resulted in three graphical formats (thermometers, vertical bar chart) and one statement format as the top choices. The use of colour to distinguish risk (red, yellow, green) and comparative information (age, sex, smoking status) were important ingredients. Consumers found formats which combined information helpful, such as colour, effect of changing behaviour on risk, or comparison with a healthy older person. GPs preferred formats that helped them relate the information about risk of CVD to their patients, and could be used to motivate patients to change behaviour.

Several formats were reported as confusing, such as a percentage risk with no contextual information, line graphs, and icons, particularly those with larger numbers.

Whilst consumers and GPs shared preferences, the use of one format for all situations was not recommended. Overall, people across groups felt that risk expressed over five years was preferable to a ten-year risk, the latter being too remote.

**Conclusions:**

Consumers and GPs shared preferences for risk representation formats. Both groups liked the option to combine formats and tailor the risk information to reflect a specific individual's risk, to maximise understanding and provide a good basis for discussion.

## Background

Communicating risk is an important ingredient in the primary prevention of cardiovascular disease (CVD), including coronary heart disease and stroke. In Australia, CVD has a substantial impact in terms of deaths, illness, disability and health system costs [1]. The majority of CVD events are preventable by addressing modifiable risk factors such as high blood pressure [2]. Because most people have more than one risk factor for CVD, overall risk of disease (absolute risk) for a given time period may be high. This can occur even when some risk factors are within normal thresholds. Therefore, effective communication between general practitioners (GPs) and consumers about individual risks and strategies to reduce risk is essential, and offers great potential in terms of lives saved, illness prevented and cost effectiveness.

In Australia, disease focused non-government organisations which share an interest in common risk factors have come together under the umbrella of the National Vascular Disease Prevention Alliance (NVDPA) [2]. The Alliance consists of representatives from the National Heart Foundation, National Stroke Foundation, Australian Kidney Foundation and Diabetes Australia. The purpose of the NVDPA is to work collectively to reduce the burden of vascular disease in Australia.

The NVDPA has been working towards an absolute risk-based approach to the prevention of vascular disease since 2003, with a focus on people aged 50 and over who are at highest risk [2]. The implementation of an absolute risk approach - incorporated into risk assessment tools for GPs - was not well understood when compared with traditional clinical practice. Traditional practice more commonly relies on treating individual risk factors once they reach a defined threshold. However, there is evidence to suggest that this approach is less effective than looking at the sum of all risk factors present in an individual.

Communication of standardised risk information between GPs and health consumers is important. However, attention to the technical challenge is fraught with the possibility of diminishing attention to the interpersonal complexities and flexibility required for individual consultations [3-5]. There is potential for confusion and misunderstanding [6]. At worst, the danger of manipulation exists [7]. How risk is communicated affects the choices that people make, such as taking up more 'risky' treatment options when information is framed positively (e.g. chances of success or survival versus chance of death) [8]. The benefits of a standardised format derived from a risk calculation tool may include accurate assessment [9,10], improvement in communication between health professionals [5] and increased public understanding. The appearance of Internet-based risk assessment tools for the public underscores the move to involving health consumers more generally in the process of risk assessment for major chronic diseases [11].

A review of the literature reveals that the challenges associated with using different risk formats are sizeable. In particular, the main issues relate to content (availability of empirical data, validity of available data for the population of interest), calculation (by clinician using tool), and the format for representation of data. Options include simple or more complex styles of communication, including presenting risks verbally or numerically, a choice of pictorial, graphic, or statement formats, framing risks (e.g. negative or positive frames), and distinguishing absolute or relative risk formats [12]. Absolute risk is defined as the observed or calculated risk or rate of an event occurring in a defined population over a specified time period, while relative risk is used to compare the risk or rate of an event in two different groups of people [13]. Fortin and colleagues [14] studied women at hypothetical risk for various diseases, including coronary heart disease, and have shown that women preferred bar graphs to line and thermometer graphs, faces (i.e. icons), or survival curves. Different formats also affect understanding [15]. Graphical presentations have been shown to be attractive to GPs, because they can convey complex information without the need for detailed explanation [4]. No research was identified that explored views of members of the public (here termed health consumers) and GPs about the same format options.

The overall aim of our research was to explore consumer and GP views and preferences about the most suitable formats for the representation and discussion of absolute risk for CVD. It was sequenced to allow different questions to build on each stage. This paper presents the combined findings from stage (i) development of formats for presentation of absolute risk for CVD; and part of stage (ii) focus groups with consumers and GPs separately to explore comprehension and preferences for risk formats for CVD. Focus group discussion of how risk should be discussed by GPs and consumers was also undertaken but is not presented here.

## Methods

### Stage (i) Development of formats

A search of databases (Medline, CINAHL, Embase) and the Internet was undertaken to compile an inventory of different absolute risk tools and formats currently in use in general practice at the time this research was undertaken (reference year 2003). Formats identified included statements (using numerical data), icons and graphical formats, alone or combined. The summary of representation formats was presented at a workshop in October 2003 of research partners, experts and government personnel, and feedback from participants and co-researchers was obtained. The agreed formats for presentation at the focus group stage were then assembled.

### Stage (ii) Conduct of focus groups

The objectives for stage (ii) were to explore comprehension of and preferences for different formats of absolute risk representation for CVD. This was assessed in terms of clarity and reported ease of understanding regarding the severity of the specified risk. Each different format presented the same risk information. Focus groups were chosen for the research method because they facilitate the expression of attitudes and feelings, enable a wide range of potentially conflicting views to be expressed in an interactive way, and enable the researcher to understand how things might 'work in practice' [16]. They may also reduce misunderstandings, which was considered a possibility in this study given the complex nature of the subject matter [17].

The study was approved by La Trobe University's Institutional Ethics Committee. The questions (developed from a review of the literature of risk communication) and formats were then piloted on volunteer university colleagues, and sent to research partners for feedback. Questions subsequently were revised to reduce the length of general discussion of health and risk.

The sampling criteria for consumers were as follows: they were aged 40-60 years and had no previous diagnosis of CVD (heart attack, angina, peripheral arterial disease or stroke). Presence of relevant risk factors, such as high blood pressure, high cholesterol, or smoking was permitted but not recorded or assessed. Recruitment strategies also aimed to include equal numbers of men and women, as well as people from a range of socio-economic circumstances. Recruitment targeted several community-based health organisations in metropolitan Melbourne and rural Victoria and continued until there were three focus groups for each participant group (two metropolitan, one rural) with at least six participants per group.

The sampling criteria for GPs were to aim for equal sexes and access to a range of relevant practice resources. These included computer- and paper-based practices, practices that included nurses, higher Medicare (national health insurance) fee entitlements, as well as both metropolitan and rural practices. If possible, GPs with a sizable caseload matching the consumer sampling criteria were encouraged. Recruitment was through Melbourne metropolitan Divisions of General Practice (two focus groups, Northern and Dandenong Divisions) and one focus group in the rural Division of the Otways, in SW Victoria.

All prospective participants were asked to volunteer their attendance, were given verbal and written information, and offered an honorarium towards costs of participating. All were asked to sign a consent form when they attended the group meeting, and completed a short demographic data sheet. As some of the participants came from small communities, all were advised that none of the data would be identifiable below the level of 'consumer' (C) or 'general practitioner' (GP).

Each group met for about 90 minutes in a place convenient to the participants, and was moderated by two researchers (JS and SR). All were taped and notes were taken. In one instance, the tape recorder failed. Notes taken during and after the session by both researchers, written up immediately and double-checked for accuracy.

The consumer focus groups commenced with a brief outline of the project objectives. This was followed by an outline of the vascular system, explanation of CVD, its causes and the multifactorial nature of risk factors, and the meaning of the term 'absolute risk'. The GP focus groups commenced with a short introduction to absolute versus relative risk concepts, the multifactorial nature of CVD, and information on risk calculation tools [see Additional file 1: Focus group information materials].

In groups, participants were asked about their understanding of risk (and absolute risk), the formats were outlined and discussed, and participants were encouraged to talk about how such tools might be discussed with the other party (the patient or the doctor, depending on the group). The purpose of these examples was to explore which formats were most easily understood and which formats accurately conveyed that the risk figure used was a high risk for CVD. For each different risk format the consumers were asked "Is it clear to you?" and "How does it make you feel (e.g. scared, concerned, reassured, don't care)?" The GPs were asked "Do you think your patient would understand?" and "How do you think your patient would react (e.g. scared, concerned, reassured, no effect)?" In addition, questions were asked about preferences for risk timeframes: risks at five compared with ten years were discussed with both groups, although formats only presented a five year option.

Finally, participants were asked to record their three top preferences and reactions to the different risk formats in a booklet, which they returned to the facilitators at the end of the session.

### Analysis of focus groups

All transcripts and notes were manually coded by JS according to the questions stated above. The coded transcripts were then organised into themes, which addressed the objectives, particularly connecting formats with comprehension and attitudes [18]. Careful attention was paid to divergent views. SH and CK checked the comments in the booklets against the draft analysis, again with attention to any divergent views. A draft report of the initial findings from the focus groups was written and circulated for comment by members of the research team and steering group, and the focus group participants. Errors of fact were revised and important omissions included.

Consumers' and GPs' top preferences for formats were tabulated. A simple summation was made of the number of preferences for each format. Following tabulation, the written responses to discussion questions as well as the transcripts of the focus groups were analysed independently and a synthesis of the emergent themes was drafted and checked by three authors (JS, SH, CK). A draft was also sent to a fourth author for comment and independent feedback on risk communication themes (AE). Data were then organised according to the research question, specifically addressing preferred formats for the representation of risk information.

## Results

### Stage (i) Development of formats

Sixteen different formats were prepared, including statements, icons (i.e. crowd figures), graphical formats, alone or in combination, and with variable use of colours [see Additional file 2: All formats]. The absolute risk equations that were used to inform the currently used risk probabilities [2] were derived from the Framingham Heart Study [19]. The Framingham Heart Study is the most widely used to assess CVD risk, and has been validated in a couple of Australian cohort studies, although there is some question over the generalisability of the findings to the broader Australian population [20]. In our present study, the final set of formats for the pilot stage presented the absolute risk of a 55 year old woman with a 16% risk of CVD in five years. According to the National Heart Foundation of Australia and Cardiac Society of Australia and New Zealand's (NHFA/CSANZ) Position Statement on Lipid Management [21], people with a five-year CVD risk of greater than or equal to 15% are considered high risk. For this study, 16% was chosen as a realistic representation of an important level of risk.

The consensus from the workshop of co-researchers and experts was that the assessment should be based on the outcomes of coronary heart disease ('heart attack' in the formats) or stroke; that all formats would present risks to five years (but with discussion of five and ten year options); that as many formats as possible should be presented; and that key variables of age, sex and smoking status would be included in some formats. This choice of data was based on the importance of giving meaningful information for consumers and clinically relevant information for GPs. One format, a pie chart, was rejected, as it was considered misleading.

Table 1 summarises the final formats agreed at the workshop and used in Stage (ii) of this research. The different formats included one or more of the following elements: the same risk expressed with different statements (using numerical data); numbers (expressed as percentages, natural frequencies and odds); icon displays; graphical formats (vertical bar chart, line graph, thermometer scale); and use of various colours (red, yellow, green, blue). The icon displays comprised large or small faces, green and smiling or red and sad, grouped in a random or uniform distribution to represent the risk. Some formats included information that allowed the individual's risk to be compared to that of a hypothetical person with either a lower average absolute risk, or the individual's risk if on treatment or if a non-smoker.

**Table 1:** Risk formats: Description of formats and summation of preferences

Option	Format	Statement, plus format explanation		Consumer format preferences (n = 19)	GP format preferences (n = 18)	Total
1	Statement	*Your risk of having a heart attack or stroke within the next 5 years is 16%*.		3	0	3

2	Statement	*Your risk of having a heart attack or stroke within the next 5 years is 16%. The average risk for someone of your age and gender is 8% in 5 years*.		3	3	6

3	Statement with icon display	*The odds that you will have a heart attack or stroke in the next 5 years are about 5 to 1 with six faces*. 5 grouped green, smiling and 1 red, sad.		2	2	4

4	Statement with icon display	*1 in 6 people like you will have a heart attack or stroke within the next 5 years with six faces*. 5 adjacent green, smiling and 1 red, sad.		1	1	2

5	Statement [36]	*You are 50 years old. Your risk of having a heart attack or stroke within the next 5 years is 16%. This is the same risk as an average person who is 67 years old!*		7	5	12

6	Line graph [37]	*CVD risk and life expectancy. Chance of surviving without having a heart attack or stroke*. Lines graphed for 'average risk profile' in blue and 'your risk profile' in red.		3	0	3

7	Icon display	*Risk shown in icon display. Your risk*: 100 faces arranged in a block, 84 grouped, green, smiling (no heart attack or stroke within 5 years) and 16 grouped, red, sad (heart attack or stroke within 5 years).		1	0	1

8	Icon display	*Your risk*: 100 faces, organised as per option 7 (84 green, 7 red). *Average risk for someone your age and gender*: 100 faces, same organisation, (92 green, 8 red).		3	2	5

9	Icon display [38]	*Your risk*: 100 faces in a block but 'risk' faces distributed at random, with 84 green, smiling (no heart attack or stroke within 5 years) and 16 red, sad (heart attack/stroke within 5 years).		2	0	2

10	Icon display [38]	*Your risk*: 100 faces, organised as per option 9 (84 green, 7 red random distribution). *Average risk for someone your age and gender*: 100 faces, same organisation, (92 green, 8 red).		1	0	1

11	Icon display	*Your risk*: 25 faces, 21 grouped, green, smiling and 4 grouped, red, sad.		0	1	1

12	Icon display	*Your risk*: 25 faces, organised as per option 11 (21 green, smiling and 4 red, sad). *Average risk for someone your age and gender*: 25 faces, as per option 11, but with 23 green and 2 red.		0	3	3

13	Thermometer display [35]	*Risk shown in a 'Thermometer'. Your risk of having a heart attack or stroke within the next 5 years*. Risk marked on a thermometer, using green for low (0-5%), yellow for moderate (5%-10%) and high (10%-20%+) in red. Arrow marks risk at 16%.		5	10	15

14	Thermometer display [35]	*Risk shown in a 'Thermometer'. Your risk of having a heart attack or stroke within the next 5 years*, and *Your risk if you were a non-smoker*. As per option 13, but with a second arrow marking risk level if a non-smoker (less than 10%).		9	15	24

15	Vertical bar chart [34]	Title: *Risk shown in bar chart*. Label for graph: *Risk of having a heart attack or stroke within the next 5 years*. *Your risk*: Red bar, at 16%; *Your risk if receiving treatment*: Blue bar, < 11%; *Average risk for someone of your age and sex*: Yellow bar, 8%.		12	10	22

16	Line graph	Title: *Risk shown in a line graph*. Label for graph: *Risk of having a heart attack or stroke within the next 5 years*. Horizontal axis: age; vertical axis: percent risk.		3	4	7

### Stage (ii) Focus groups

Three focus groups with consumers and three with GPs were held (both groups involved one each in inner and outer metropolitan Melbourne areas and one in rural Victoria).

Nineteen consumers and 18 GPs participated. The average age of consumers was 50 years whilst that of GPs was 48 years. Of the 19 consumers, 12 were women; of the 18 GPs, 4 were women. Eleven of the GPs employed practice nurses and all used computers in their practices.

Consumers with a range of different education levels were recruited; all were from English-speaking backgrounds. Five of the GPs came from non-English speaking backgrounds and stated that they sometimes used their language of origin when consulting with patients. Asked about their prior experience with risk calculators, none of the consumers had previously used a risk calculator and neither had their GPs.

### Preferred formats: overall

Table 1 summarises all formats in the order presented, with tallied preferences for consumers, GPs and total. The most preferred formats were those considered the most easily understood and the most effective in convincing consumers that 16% was a high risk for CVD over five years.

**Figure 1:**
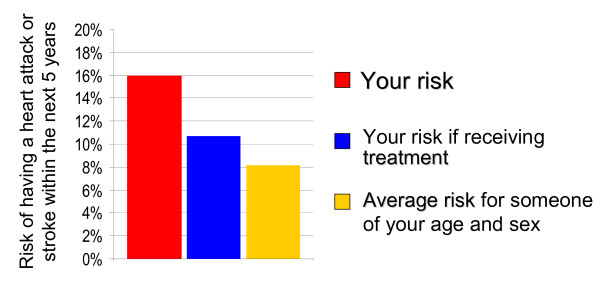
**Option 15 **[34]. - Bar chart, three bars, comparing individual's risk (red bar, highest) to risk if receiving treatment (blue bar) and to risk of someone of the same age and sex (yellow, lowest): 22 'votes'.

**Figure 2:**
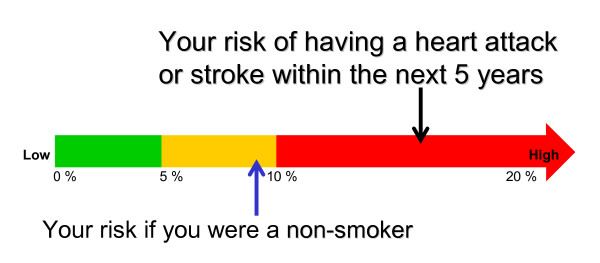
**Option 14 **[35]. - Thermometer, showing individual's risk (high, red marking) compared with risk for non-smoker (yellow): 24 'votes'.

**Figure 3:**
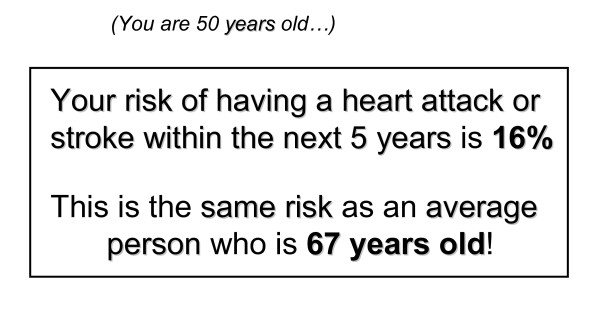
**Option 5 **[36]. - Two statements illustrating that the individual's risk is comparable to the average 67 year old: 12 'votes'.

The three formats with the most 'votes' from consumers were:

• Option 15 (Figure 1). Bar chart, three bars, comparing individual's risk (red bar, highest) to risk if receiving treatment (blue bar) and to risk of someone of the same age and sex (yellow, lowest): 12 'votes';

• Option 14 (Figure 2). Thermometer, showing individual's risk (high, red marking) compared with risk for non-smoker (yellow): 9 'votes';

• Option 5 (Figure 3). Two statements illustrating that the individual's risk is comparable to the average 67 year old: 7 'votes'.

**Figure 4:**
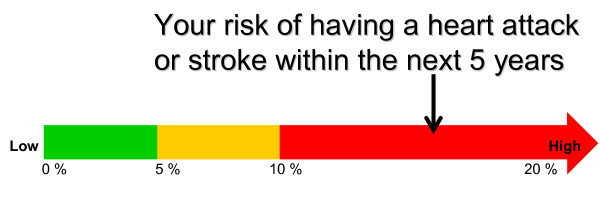
**Option 13 **[35]. - Thermometer, showing individual's risk (high, red marking): 15 'votes'.

The three formats with the most 'votes' from GPs were:

• Option 14. Thermometer: 15 'votes';

• Option 13 (Figure 4). Thermometer, showing individual's risk (high, red marking): 10 'votes';

• Option 15. Bar chart: 10 'votes'.

If the results for the top four formats are selected, consumers and GPs preferred the same four formats, listed as follows in order of preference: Options 14, 15, 13 and 5.

Importantly, all but two formats (11, 12; those with 25-50 icon combinations) received at least one 'vote' from consumers. The results for consumers varied widely, with divergent views on some formats. GPs were more consistent within their group and five formats received no 'votes' (1, 6, 7, 9, 10; statement, line graph, 100-200 icon combinations).

### Reasons for preferences

Consumers [notation 'C' in quotes] found the formats which combined information helpful in explaining and contextualising the risk:

Because of the colours and it actually explained things to me proper ... [option] 15, that made me more scared I've got more chance of having a heart attack. [C]

Where it says your risk is the same as a 67 year old, it would suddenly make you think 'oh my body is so much older'. [Option 5] [C]

Similarly, GPs [notation 'GP' in quotes] liked the formats that helped them relate the information to the patient, gave comparative information to situate the risk, and looked for information to help with motivating patients. For these reasons, Option 14--the thermometer with risk for a non-smoker compared to a smoker--was considered to be personalised, easy to understand and effectively conveyed the high risk to patients:

People are interested in themselves, it's about them, it's not about them in the population, it's about them on that day and how it's actually going to affect them at the time. [GP]

Although the bar chart (Option 15) rated well, some GPs felt that it was too complicated, and would take too long to explain. When discussing whether there were some formats that they would never use, several GPs said that they would never exclude all of the options.

Where a person has been back 16 times and you haven't had any luck you try different things every time. You just can't keep repeating yourself. [GP]

This sentiment was echoed by a consumer, who felt that different formats might be needed for different people:

I think a combination of some of those would be good because ... different ways of presenting it here will hit home more than others ... you would think a combination of two or three would probably be better than, 'here's a graph' or 'here's a thermometer' [option 13] or 'here's a bunch of smiley faces.' [C]

### Understanding the severity of the risk

When 16% was used as the sole presentation of risk, most consumer participants assumed this was safe because it was not 75% or 80%.

I don't have to worry; it's only 16%, that's OK. [C]

It was too confusing and what does it really mean, 16% of a risk, well that to me is too low, I wouldn't do anything about that. If it said 75%, that's different. [C]

It was not until they received the additional information, as was indicated in Options 5, 13 or 15, that 16% was in fact a large risk, that they became concerned.

If you are saying that in medical terminology 16% is something which is extremely high ... Well I think that is going to cause too much confusion because the general public will believe that's a low risk, so they need to be very careful about their terminology and the numbers and percentage they use. Because we aren't trained in the medical field. [C]

I think 16%, yeah, that's fine, but if the average risk is half that for other people, I thought twice the risk of the average person in my age group, and I thought, oh gosh. [C]

The use of colour was a critical component of conveying the severity of risk. Although it could be misleading it was quite powerful and familiar to all consumers:

I thought the thermometer was really good actually, definitely my top three because of the red. [C]

The use of red in the thermometer, combined with its simplicity, would mean that the patient would "feel as if they are in the danger zone." [GP]

GPs thought that the use of colour was important in conveying to patients whether something was 'good' or 'bad'. When asked if they thought the colours made a difference, participants equated it with the use of colour in traffic lights.

Yes. Absolutely red is bad. Green is OK, green is good. Yellow is good. Let's go simple; it's a stoplight isn't it? [GP]

However, some GPs thought that this could be deceptive and that people would not really understand their risk. It was suggested that risk might be represented by a graduation of colour, as in the thermometer formats (Options 13 and 14). Consumers felt similarly that the use of colour was not always straightforward. Some consumers, when seeing a mass of green faces or green on a diagram, assumed or identified with being in the green part and therefore not at risk or in danger. In other words, the colour green indicated to people that it was safe. But on a number of the risk formats the green was not absolutely safe.

Well I'm not thinking I'm one of the red people, I think I'm the green. [C]

When the green accounted for a smaller proportion of the format and there were cue words, as in option 13 or 14, people were more inclined to be concerned.

I'm worried by this, because I'm a fair way up that arrow ... scared, it really impacts, I feel urgency especially being in the high red zone. [C]

Concern was also expressed by consumer participants that a reliance on colours to communicate risk might not be successfully interpreted by colour-blind people.

### Formats which were misleading or hard to understand

The icon displays received a broad range of responses. Several participants liked the icon displays with smaller numbers of faces; however the formats with larger numbers were frequently rated as confusing or unclear.

I like the graph because it makes it look like there's something that can be done, whereas I think the faces are all alike, it doesn't look too bad ... [C]

The smiley faces to me make it look very, well it's OK, you are either in the red or you're not and even if you are, there are not many people in that. [C]

Only half the consumers understood that they were at increased risk when viewing the icon displays. The other half of the participants were confused by the format or thought that they were 'safe.' Whilst several people did like the randomly grouped red and green faces, others felt annoyed by them.

I liked the random one [option10] because then it made me think that this thing can happen randomly whereas when it's this one [option 8] I'm thinking there's just precious little chance that I'm going to fall into that red line there. [C]

A number of GPs thought that the use of 'statistics', i.e., numerical percentages, ratios and proportions, was too difficult for some of their patients to understand, or that their use might cause patients to be sceptical of their meaning. The criticism of Option 1 (single statement) was that the patient might have "no idea of what 16% means" [GP]. These difficulties in understanding the significance of percentages were seen to be in addition to the difficulties that patients may have in understanding medical conditions, such as heart attack or stroke.

Although many GPs thought that their patients would understand the icon displays, many did not believe that their patients would realise the extent of their risk if presented with these formats. GPs thought that they would have to contextualise what the tool was displaying in order for it to have impact. For example, one GP said he would say "think of yourself and five other friends, and one of you is going to have a stroke in the next five years". This made the message more powerful. The options where green and red icons were randomly displayed were found to be particularly confusing.

Some GPs indicated that they felt their patients did not respond well to numbers or charts.

I find people don't really respond very well to having figures and risks and charts and things ... the average person often chickens out when I start talking graphs and numbers and charts. [GP]

### Preference for risk timeframe: five years or ten years

Although all formats presented five year risk, participants were asked whether they were interested in knowing their risk in ten years. Most consumers said that they preferred knowing their CVD risk within five years. Risk within ten years was seen as being too far away, that it would be an excuse to put off making any lifestyle changes, or that it would not be relevant after a certain age.

I like to see what my risk is and I'd like to know before the five years is up so I can do something about it. [C]

However, some felt that a ten year risk may be more dramatic and persuasive, whereas another felt that it would give time to plan change.

An assessment over the next ten years, I reckon it would give me more chance to plan if I had to make significant changes. [C]

Several GPs thought that five years was a suitable length of time over which risk of heart attack or stroke could be calculated. Because risk increases as a patient ages, the risk calculation becomes bigger, is "too remote" and in some cases, meaningless if calculated over a long time period. Some GPs even suggested that CVD risk calculated over one or two years would be the most suitable. Some GPs wanted to use the length of time that would most motivate the patient to reduce their risk. It was also important that the GP spent time introducing the idea to the patient.

Whatever is the more impressive risk ... You've got to have something visual they can see and get a handle on. [GP]

### Terms to describe absolute risk

Most consumers had not previously heard the term 'absolute risk'. They tried to understand what the term absolute risk meant when it was explained to them and how it could be relevant to them.

When you look at it that way, you may take that on board a little more for yourself rather than think, oh well, it doesn't apply to me, I don't need to worry. But if it's related to you ... you will own it. [C]

Consumers thought it should not be called absolute risk, but rather suggested "actual", "personalised", "individual", "verified", or "calculated" risk. They suggested that these words more directly described the meaning of risk and would therefore be more likely to be taken on board, owned and understood.

I understand what you're saying I think but it is confusing ... actual is better, but if you want to have something that's more relevant to an individual then I think something along the lines of personalised or individual or something like that. [C]

Personalised, I think that might hit home more than absolute. Absolute has strange connotations about what absolute could mean. [C]

Or even verified, it's been verified, you have it like an experiment, a quantity of evidence that these things are a risk. [C]

Consumers understood 'absolute' to mean 'complete'. They commented this was not a true representation of the information they were being given about what absolute risk meant to them.

Because it seems to me it isn't really absolute risk because we all know that even given all that, there's some element of unknown environmental effects, whatever. [C]

Overall, GPs reported that they preferred to use discussion, rather than risk calculation tools, to communicate information regarding risk to their patients. In discussions about the limitations of the tool, issues associated with the choice of absolute versus relative risk became apparent. Some GPs suggested that relative risk was more visually powerful and therefore better in terms of encouraging patients to change their behaviour. Absolute risk, in comparison, was said to become "too high" and not useful as the patient became older.

I actually prefer relative risk in a way because as you get older your absolute risk gets higher and higher, and I think everyone thinks of themselves as being relative to their peers, not relative to a 35-year-old healthy person. If their risk is up on 95 and then fifth in line for the next cardiac event and all their peers are below them, that's a very simple message. [GP]

## Discussion

Consumer and GP focus group participants shared similar preferences for absolute risk formats in terms of clarity of the information, how the information personalised the risk, and how convincing the formats were in relation to the severity of the risk. We do not place undue weight on the result of tallying the votes but it is noteworthy that there was broad concordance between the two sets of participants, and that the reasons they gave for their preferences were similar. It is important to stress, though, that many of the formats were rated as clear and effective by some but unclear and confusing by others, thus presenting considerable challenges for GPs in interpreting risk data to patients.

Consistent with other research, graphical formats were perceived as helpful for representing risk [12]. Three of the four most preferred formats were in graphical format: they included a vertical bar chart and thermometer scale, with risk given in percentages, and making strong use of colour to indicate risk levels. Two also provided comparative information to help indicate degree of risk. The fourth presented a statement with risk as a percentage and comparison with an average older person. It is a challenge to place these results in context with other studies. We did not find any similar study where consumers and GPs assessed the same data. We report subtle differences with other authors [14,22,23], in particular, that preferences included both the bar chart, as well as the thermometer scale. A recent focus group study with UK consumers examining different risk communication formats and media for 10-year CVD risk [24] found similar preferences, i.e. simple visual formats, with logical use of colour and comparative cues.

The focus groups enabled us to explore the reasons why different formats successfully conveyed 16% as high absolute risk of CVD over five years. These included the use of colours to emphasise the severity of risk, and the addition of relatively simple comparative information in the thermometer scale, bar charts, or phrase, such as age, effect of changing behaviour or receiving treatment. These formats, whilst preferred, were not without warnings. Participants warned of miscommunication for people with colour blindness, or the misuse of green to indicate safety rather than neutrality. Participants also warned of placing undue emphasis on a small number of specific formats and suggested that variation might be helpful depending on the patient, a finding in line with other focus group research with GPs [4].

The consumer and GP participants also had similar risk formats that they identified as less favoured. Many, although not all, did not think the icons and the line graphs were clear or that they indicated high risk. These formats were often rated as confusing or potentially misleading, particularly icon displays with a larger number of faces, a finding replicated by others [24]. It is possible that the effect of formatting the risk in natural frequencies in the icon formats (e.g. '1 in 5 people ..'), recommended by risk communication experts [6], was diluted or adversely affected by the use of icons.

The ability to use the formats to personalise the risk (i.e. viewed from either consumer or GP perspective) also received support. The data indicates a bridge that needs to be built between the statistical and technical concepts inherent in an absolute risk tool with the communication between doctor and patient. Consumers did not like the term 'absolute' and favoured labels, such as personalised, that linked the risk more closely back to them. GPs looked for information that would link to the patient and allow them to place the risk in the context of the patient's life. This may be important in terms of influencing behaviour change [8,22,25] although recent research on the effects of risk communication in the context of screening identifies little impact of a calculation of personalised risk *per se *[8].

Not only was the referential context important (i.e. in relation to the risk) but the delivery of the absolute risk data needed to be placed in the context of communication between patients and their GPs [26]. Both parties emphasised the role of communication as the basis of good medical care. Doctors' understanding of the concepts of absolute risk, their confidence to communicate it, and the ability to use the tools effectively, will also require attention and education [27]. In this context, the first key element of communication in the focus group was that 16% risk represented a high risk but this was not well understood by the consumers. Examining the implications of the findings, one question is whether different formats are therefore a secondary consideration and patient education about risk the primary issue. This argument could be taken further to reflect on whether the research sought persuasive formats rather than formats that informed people. In defence of the method, the results provide evidence that both sets of responses distinguished clear from confusing or ambiguous formats, and respondents also noted those formats with contextual cues to facilitate good discussion and understanding of risk. These findings are important because the identified discord between perceptions of risk among health professionals and patients may be a fundamental issue to address before communication of risk and shared decision-making about long-term preventative treatments. This is an area where further research is needed.

Both consumers and GPs, in the main, preferred the five-year risk time frame. However, there is an element of bias as the risk was presented to the focus groups in terms of five years and may have seemed to promote this time frame. A ten-year time frame was considered too remote and therefore unhelpful in promoting behaviour change or appreciating the degree of risk [2]. However, this time frame is consistent with the Heart to Heart decision aid [28], which has shown promising effects in a pilot randomised trial [29]. GPs were willing to consider any information that could help them motivate their patients, and this was one reason for not rejecting any of the presentations. The range of preferences elicited by participants, with several comments supporting the ten-year time frame, suggests that a common approach may not be suitable in all cases. It is similarly difficult to place these results in context with other research, which has used a ten-year timeframe for coronary heart disease [23]. Thus, this is another area where further research would be helpful.

Several formats were misleading or confusing, suggesting that some formats could be unhelpful at best and harmful at worst [30]. For instance, the severity of a risk of 16% was not apparent as a high risk to people without contextual information such as colour and comparison with other 'healthier' figures. Whilst preferred, the bar chart was considered complex by some GPs. Use of statistics could also be problematic for some patients, with GPs highlighting the need for patients to comprehend other important information about the disease itself.

### Strengths and weaknesses of the study

To our knowledge this is the first study to describe the views of consumers and GPs about risk communication formats. We successfully recruited participants within our target populations representing both metropolitan and rural constituencies. Use of qualitative techniques provided an appropriate method for eliciting the various perspectives but using a standardised approach.

Potential limitations include that the number of focus groups was small, and participants may have self-selected: GPs with an interest in risk communication, possibly with preferences already formed, may have attended. The groups had unequal numbers of males and females. Some consumers had difficulty understanding the risk information. All consumers were discussing hypothetical scenarios, whereas GPs were drawing from their experience, although not necessarily with risk tools. Group-formed preferences for particular formats may have emerged through the dynamic of each group. In addition, the format options were always presented in order from 1-16, so a possible bias toward the formats at the beginning and the end of the list must be considered.

We did not test understanding. Rather, comprehension was explored solely through interaction and the noting of preferred formats. It is possible that the research could be construed as manipulative, with a danger that we did not provide people with enough formats to explore the variable use of numerical data, for example, presenting risk in natural frequencies without using icons [6]. This might have been possible if we had conducted a larger number of focus groups, and sequenced them according to emerging format preferences. However, a strength of the study was that all formats presented the same risk and all participants knew the risk was considered a high figure, and so assessed the formats on that basis. Finally, the concordance between the consumers and GPs may improve the plausibility of the findings, although we do not go as far as suggesting that they are generalisable [31].

### Implications for further research, policy, and practice

The findings raise issues for primary prevention public health campaigns involving communication and assessment of absolute risk for CVD. Whilst many consumers were aware of various risks for heart attack and stroke, none had heard the term 'absolute risk'. Many people preferred to replace the word 'absolute' with a more descriptive word such as 'personalised'. This suggests that well-informed consumers, with an interest in the evidence base of health information, will seek additional information and might challenge the source and use of the epidemiological data [32]. GPs wanted tools that could help them motivate patients to change their behaviour and hence some preferred relative risk formats (which were not the subject of this research). Communicating a risk derived from population studies to an individual was the challenge.

Whilst consumers and GPs had similar views on the most effective and accessible formats (in terms of the intention of the risk information), the range of preferences, and the comments against limiting options, suggest that tools could be developed with various format options. These could be tested with wider samples of GPs and consumers. Concordantly, it has been shown that involving consumers in the development of health information materials improves knowledge and understanding [33].

We should not assume there is a 'magic bullet' for communicating risk to all consumers. However, risk assessment and communication is likely to become more prevalent across diseases. It is important that healthcare professionals are consistent in their assessment of risk but use a range of preferred formats to tailor and communicate - to improve understanding, and motivate change [26]. These results overall suggest avenues for further research into the effectiveness of different risk representation formats in facilitating understanding, behaviour change and improved health outcomes.

## Conclusions

Risk assessment is a valuable tool for GPs, particularly in the context of illnesses such as CVD which are often highly preventable. However, there are many pitfalls which may be encountered in communicating risk assessment to individual health care consumers. We believe this to be the first study to collect the viewpoints of both GPs and consumers about the same set of formats for risk representation. Our qualitative findings that overall both groups prefer similar formats seems to reflect the clarity and suitability of these formats. However, the relationship between the GP and the consumer is still critically important to ensure that the consumer's perceived risk is in fact accurate, and to frame this risk in the context of motivation for behaviour change or other relevant outcomes. Many of the formats of risk representation are valuable healthcare tools, and having several formats available increases the ability to tailor discussions to facilitate consumer understanding. Most importantly, the format used must not be misleading, and this study was able to identify several formats which were usually effective for many people. Of note, however, there were no unanimous votes for or against any of the risk representation formats. Risk communication will continue be refined and more research is needed into its effectiveness.

## Competing interests

The authors declare that they have no competing financial interests. Andrew Tonkin has been a member of Australia's National Vascular Disease Prevention Alliance and Dominique Cadilhac is currently a member.

## Authors' contributions

SH directed the study, conducted the analysis, and wrote drafts. JS conducted the focus groups, conducted analysis, and wrote drafts. DC contributed to the selection of formats and contributed to drafts. AE analysed data and commented on drafts. CK analysed data and wrote drafts. SR conducted literature review, selected formats, and conducted focus groups. RR conducted literature review and commented on drafts. AT initiated the study, contributed to the design, and selection of formats. All authors read and approved the final manuscript.

## Author information

Dr Dominique Cadilhac, Honorary positions:

Honorary Research Fellow, The University of Melbourne, Victoria, Australia

Honorary Research Fellow, Deakin University, Victoria, Australia

National Stroke Foundation, Australia

Whilst conducting the analysis, Carrie Kaufman was with the University of Massachusetts Medical School, Worcester, Mass., USA.

## Pre-publication history

The pre-publication history for this paper can be accessed here:

http://www.biomedcentral.com/1471-2458/10/108/prepub

## Supplementary Material

Additional file 1 **Focus group information materials**. The materials contain the information packages given to either consumers or GPs at the start of the focus groups. Click here for file

Additional file 2 **All formats**. All sixteen formats in the order presented at the focus groups. Click here for file
