# Supplementary material for: Absolute risk representation in cardiovascular disease prevention: comprehension and preferences of health care consumers and general practitioners involved in a focus group study
Source: BMC Public Health. 2010 Mar 4;10:108. doi: 10.1186/1471-2458-10-108 (PMC2845101; doi:10.1186/1471-2458-10-108)

## Additional file 1: Focus group information materials

## **Consumer information (excluding formats)**


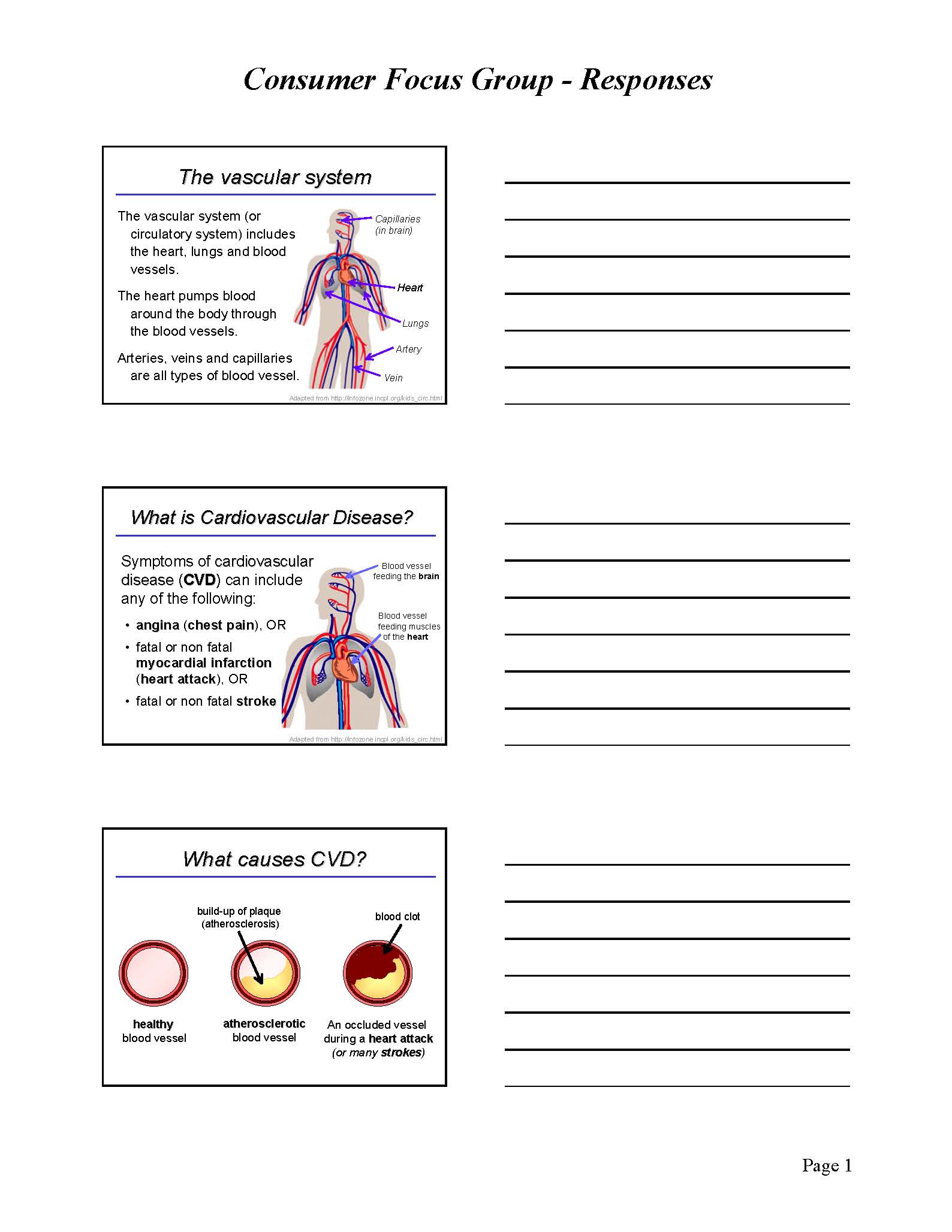


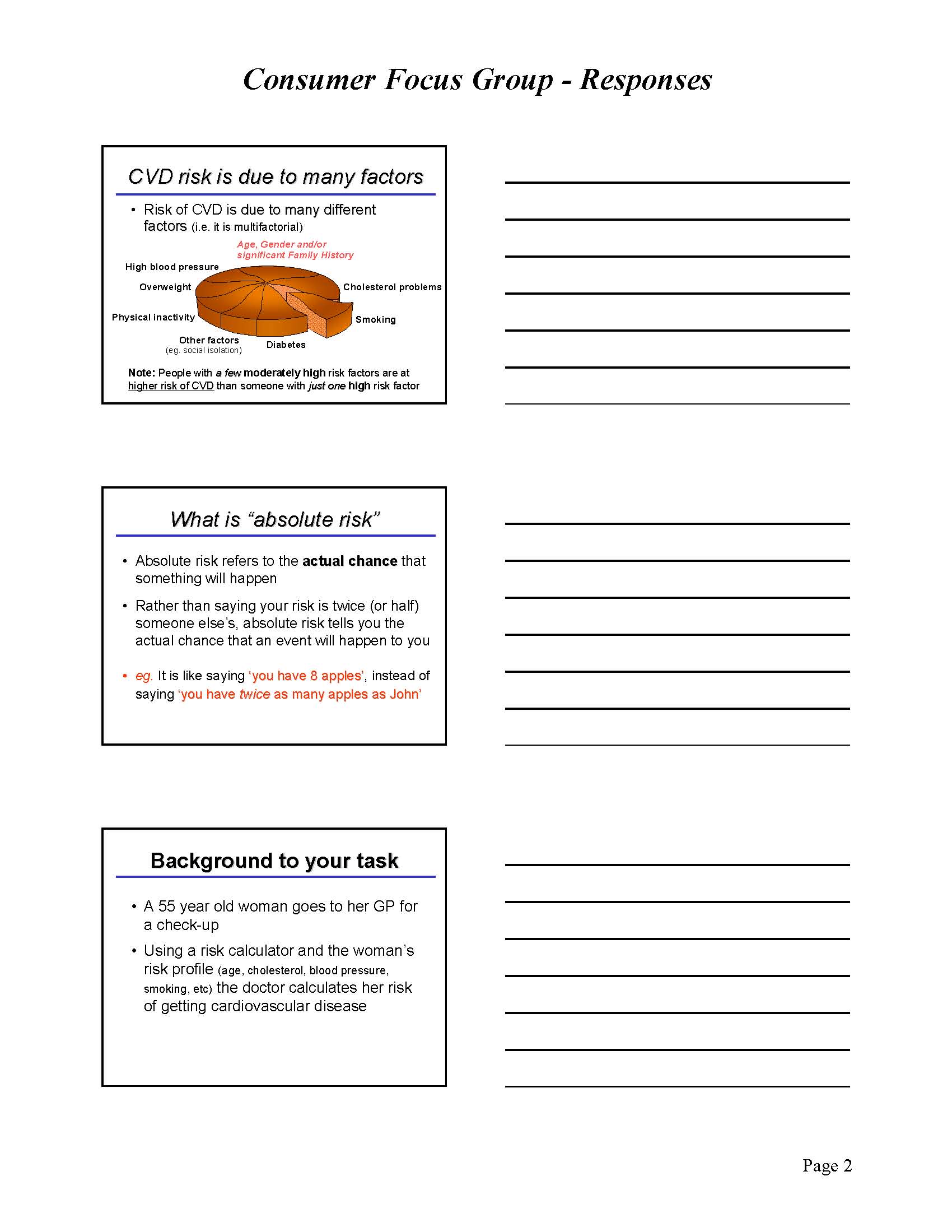


**GP information (excluding formats)**


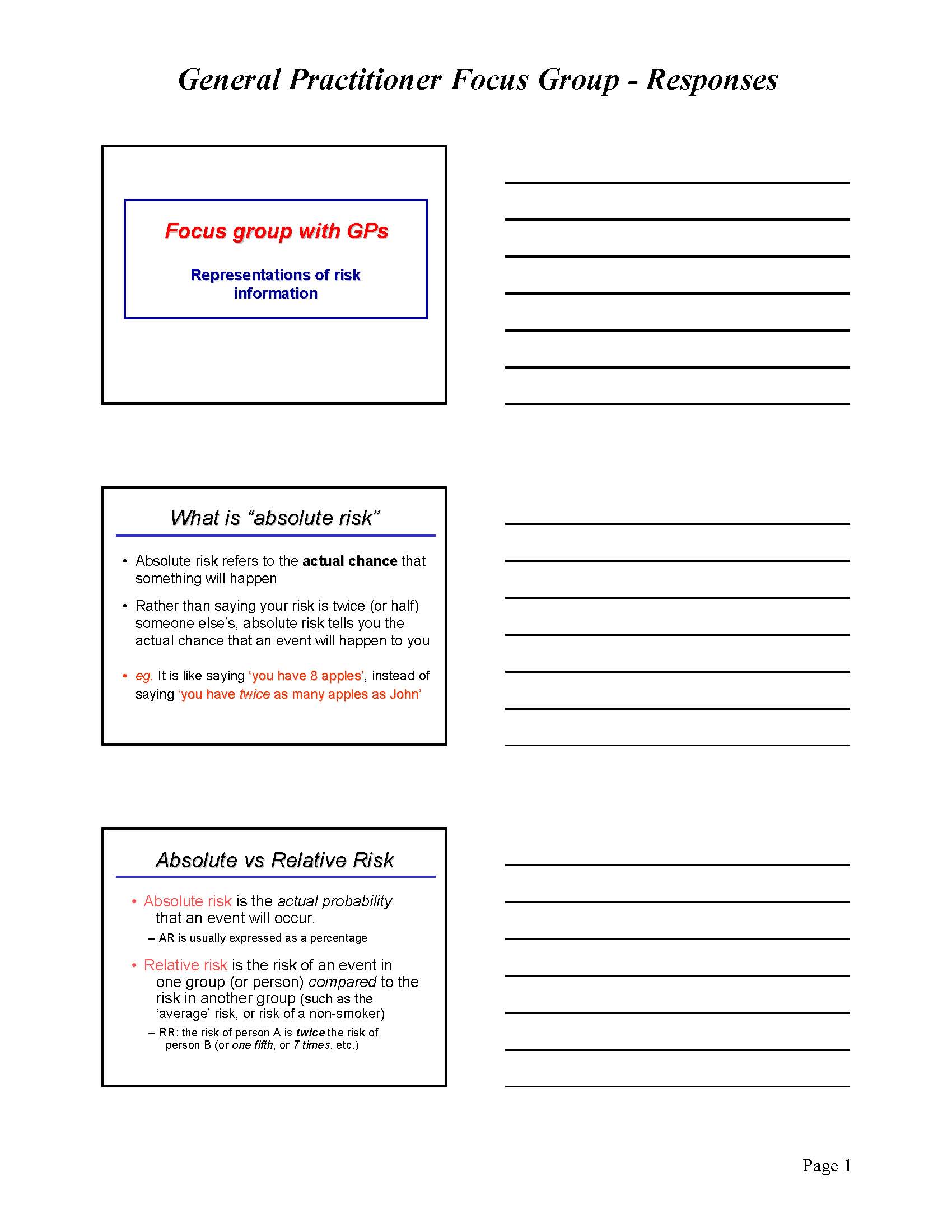


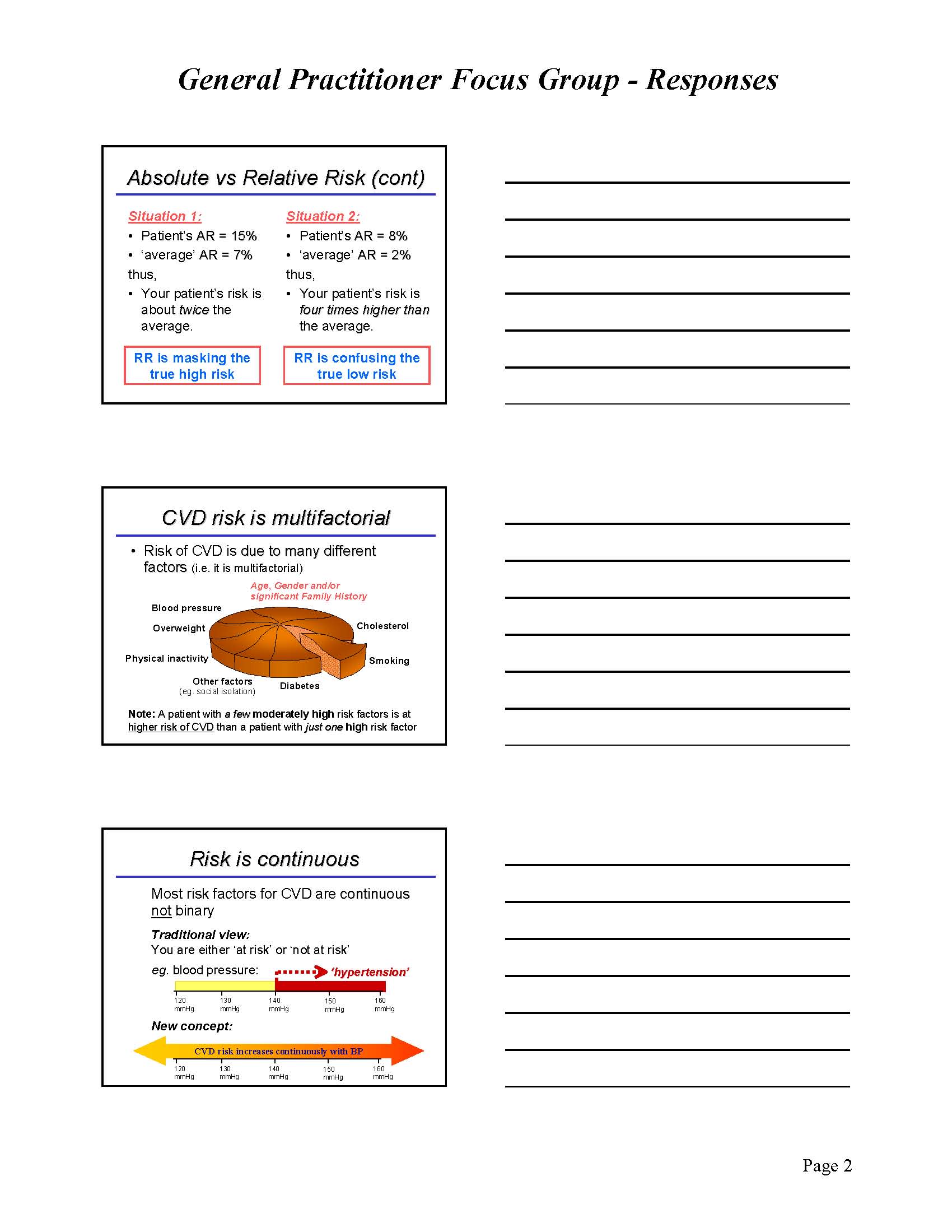


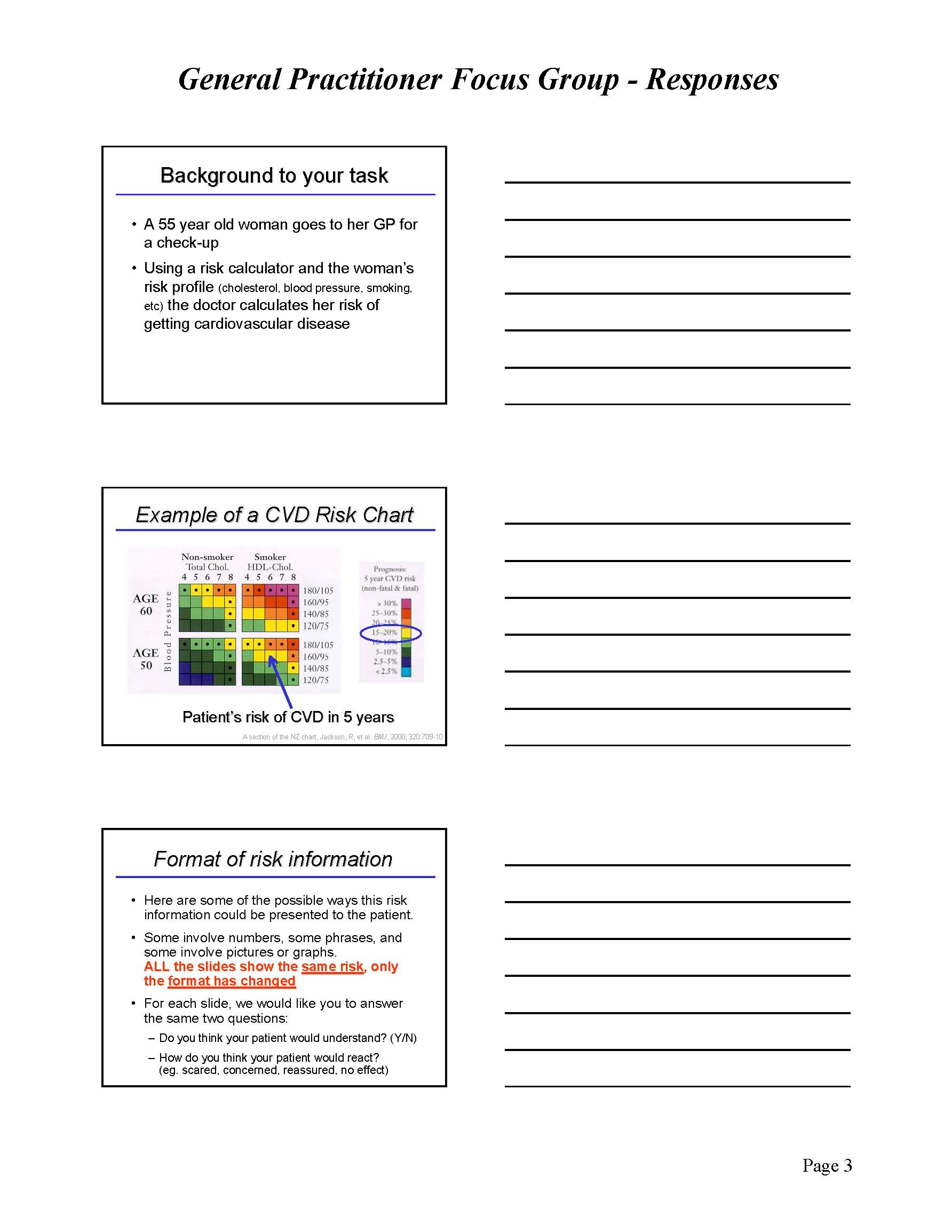

Supplement: Additional file 1 — Focus group information materials. The materials contain the information packages given to either consumers or GPs at the start of the focus groups. [file 1471-2458-10-108-S1.DOC]
