# Supplementary material for: Absolute risk representation in cardiovascular disease prevention: comprehension and preferences of health care consumers and general practitioners involved in a focus group study
Source: BMC Public Health. 2010 Mar 4;10:108. doi: 10.1186/1471-2458-10-108 (PMC2845101; doi:10.1186/1471-2458-10-108)
Supplement: Additional file 2 — All formats. All sixteen formats in the order presented at the focus groups. [file 1471-2458-10-108-S2.DOC]

**Additional file 2: All formats**


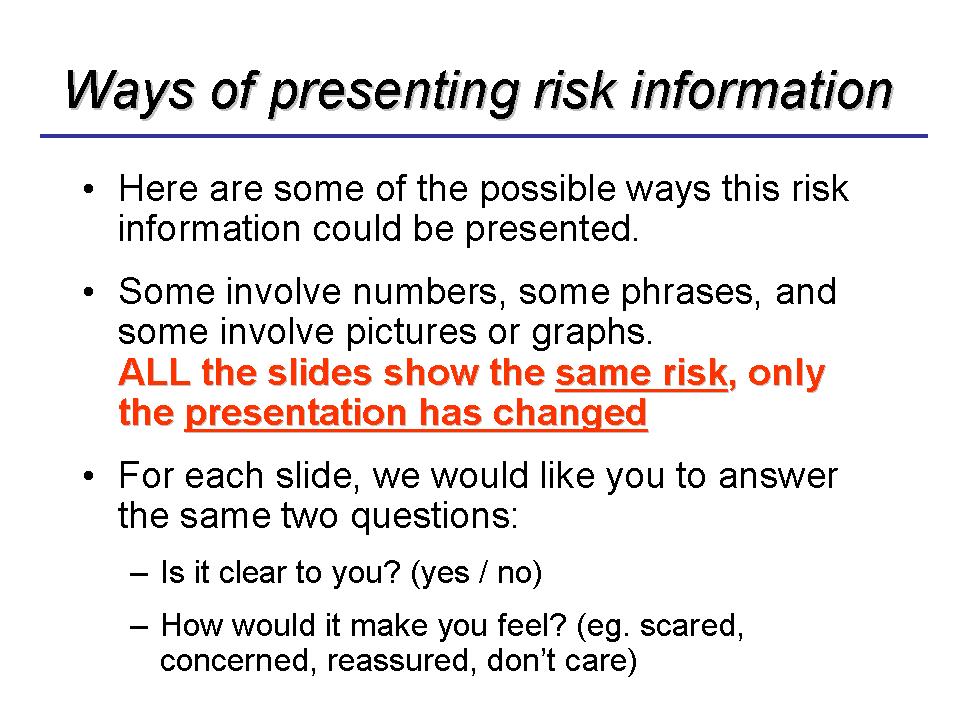


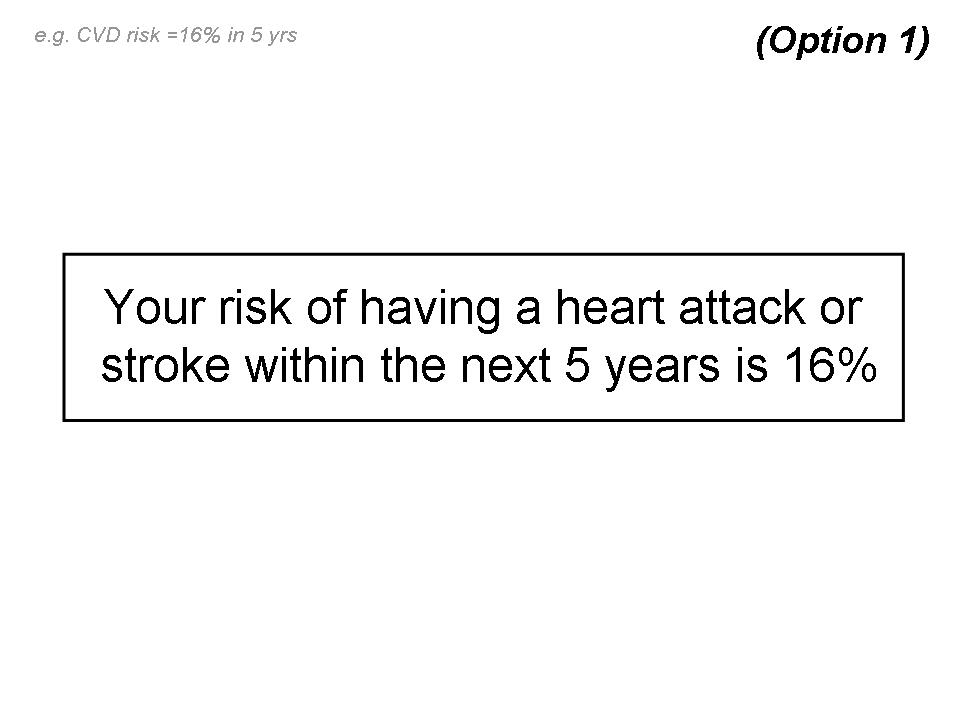


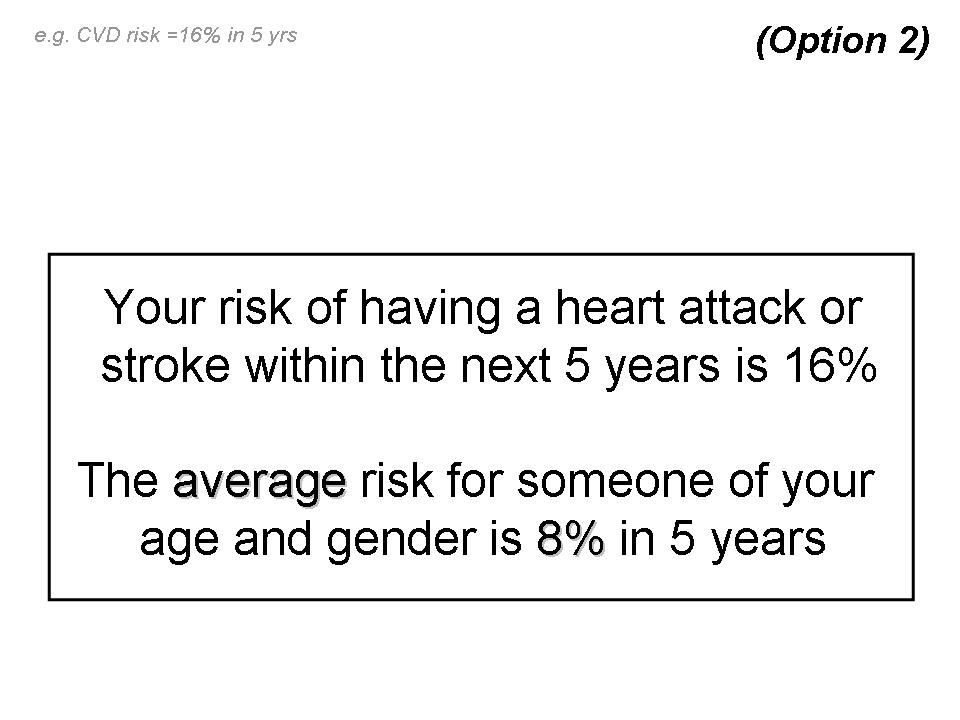


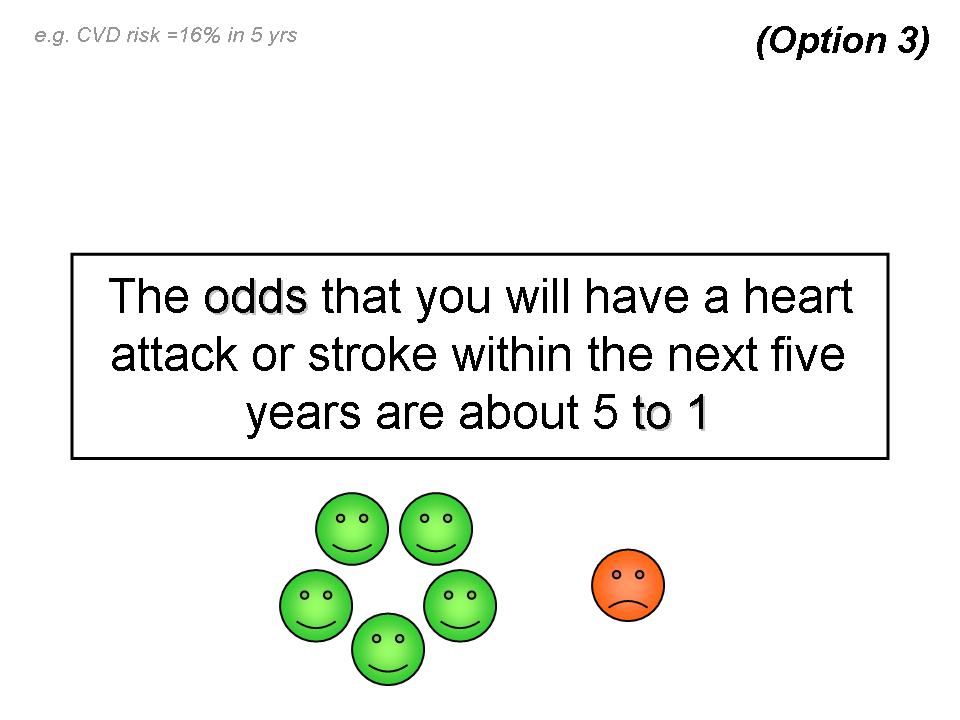


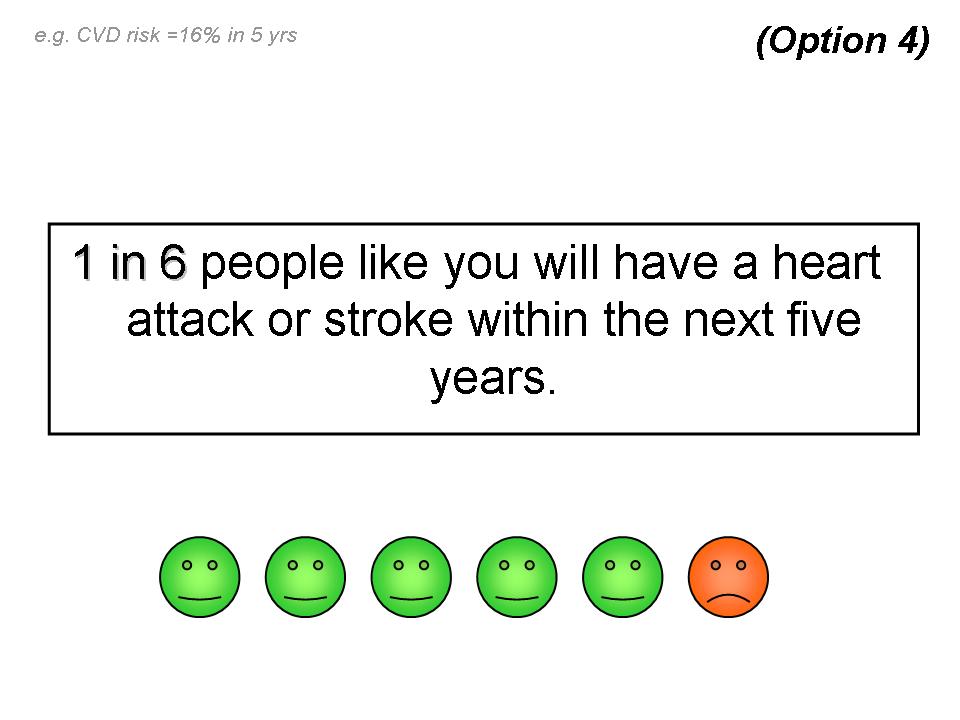


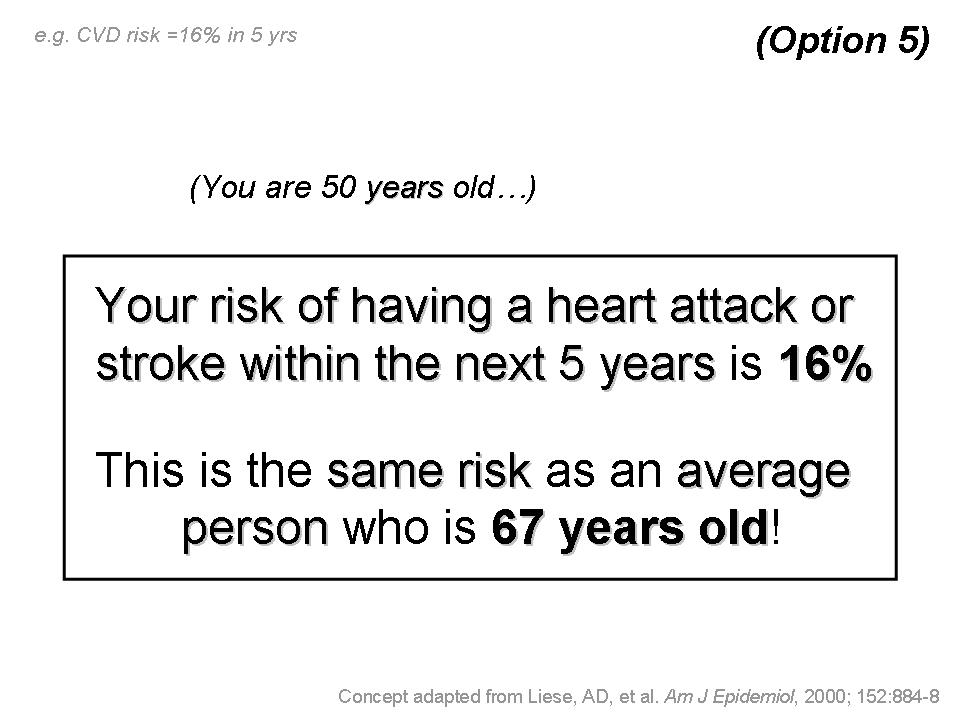


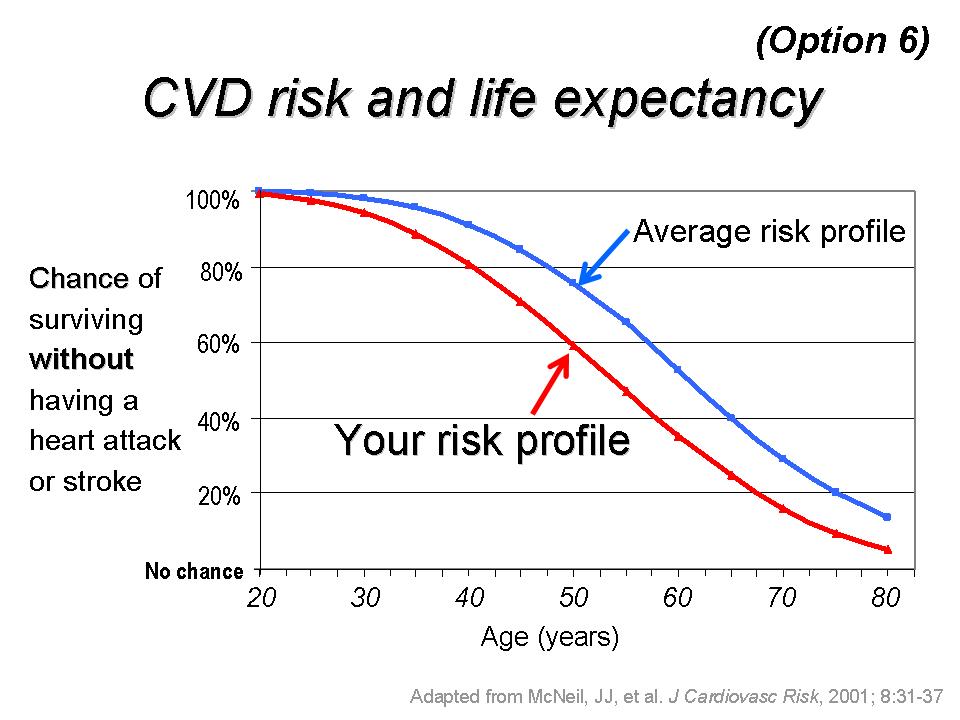


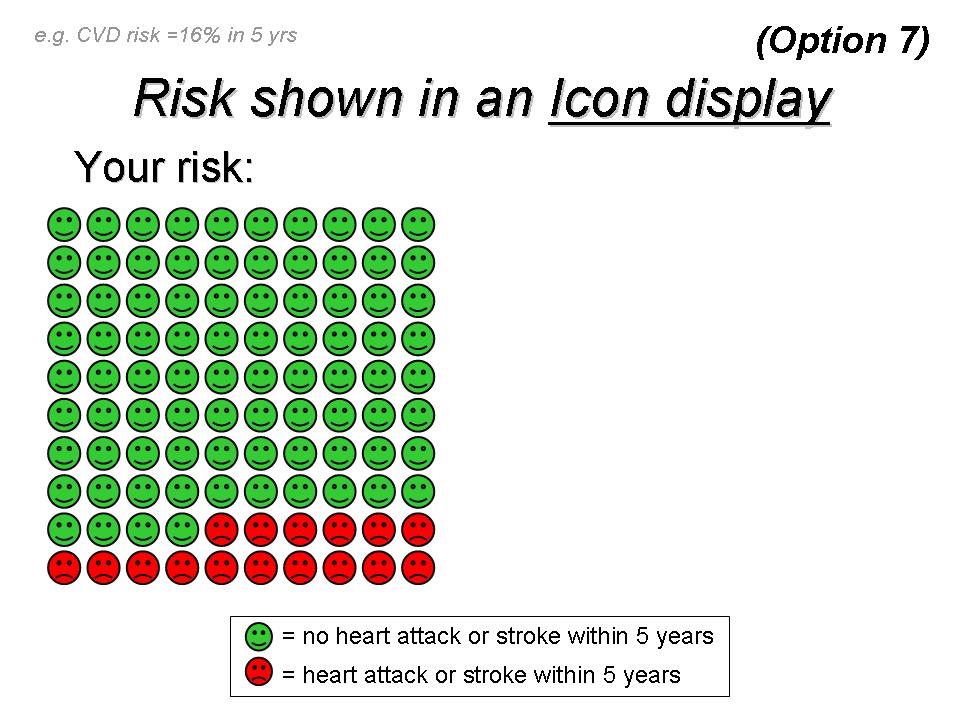


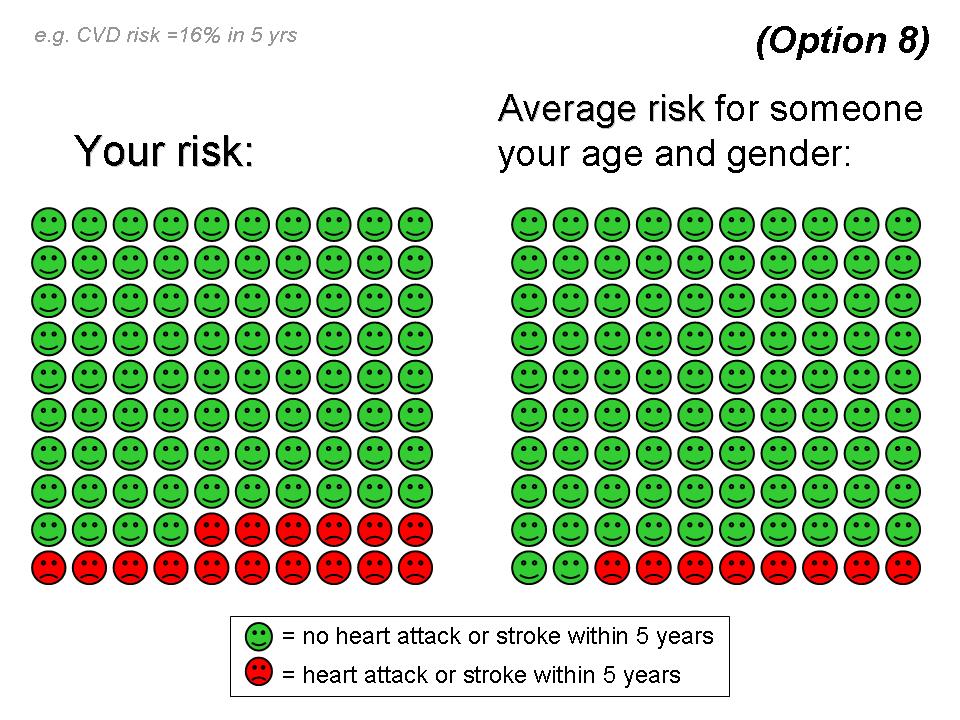


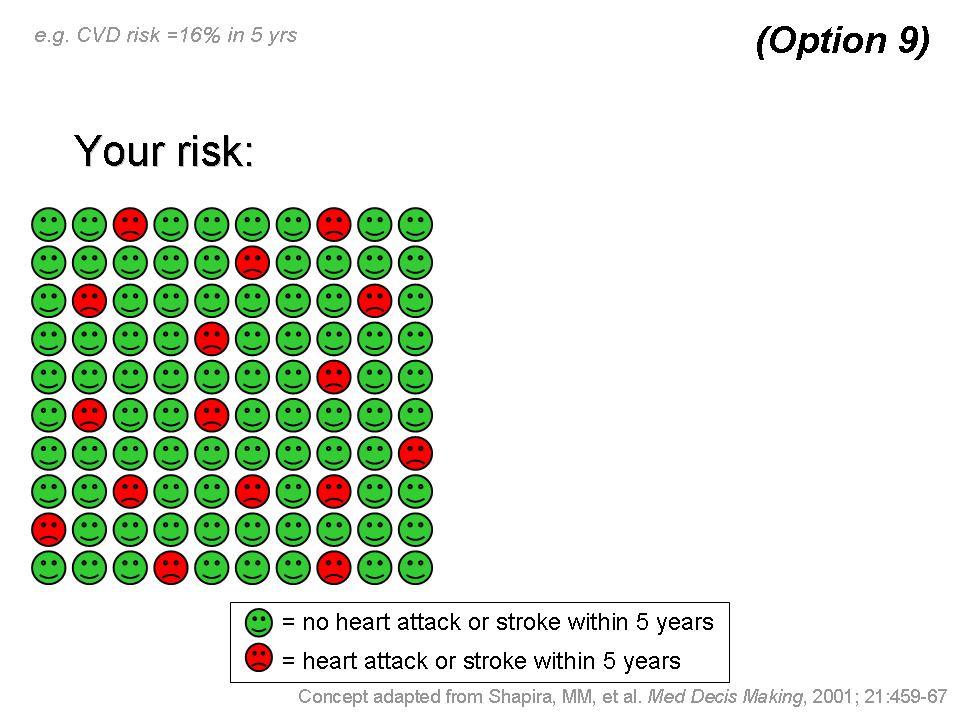


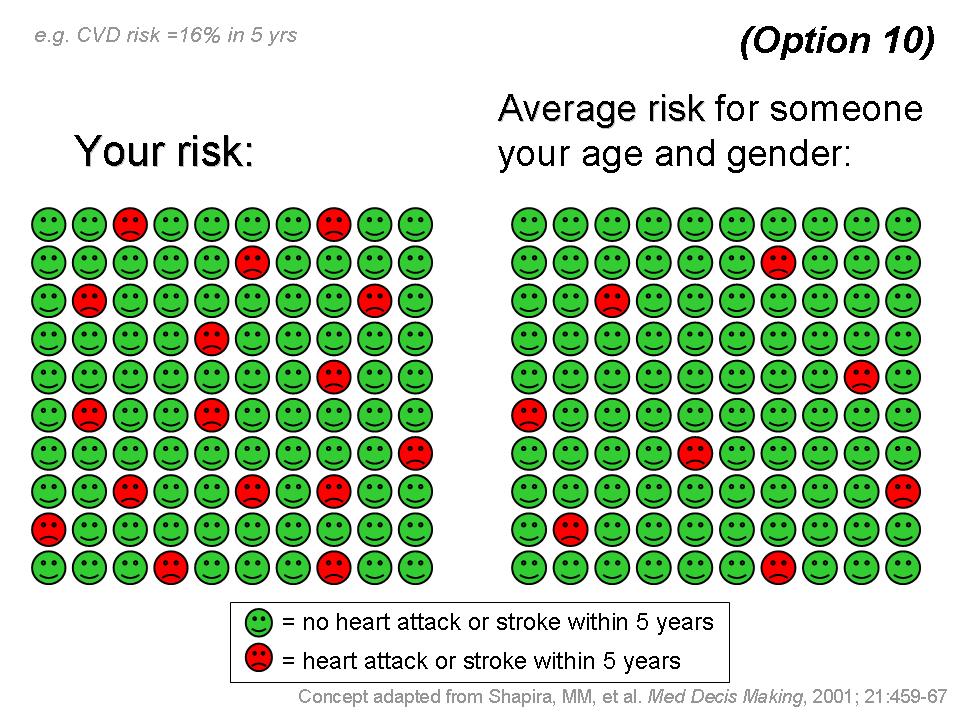


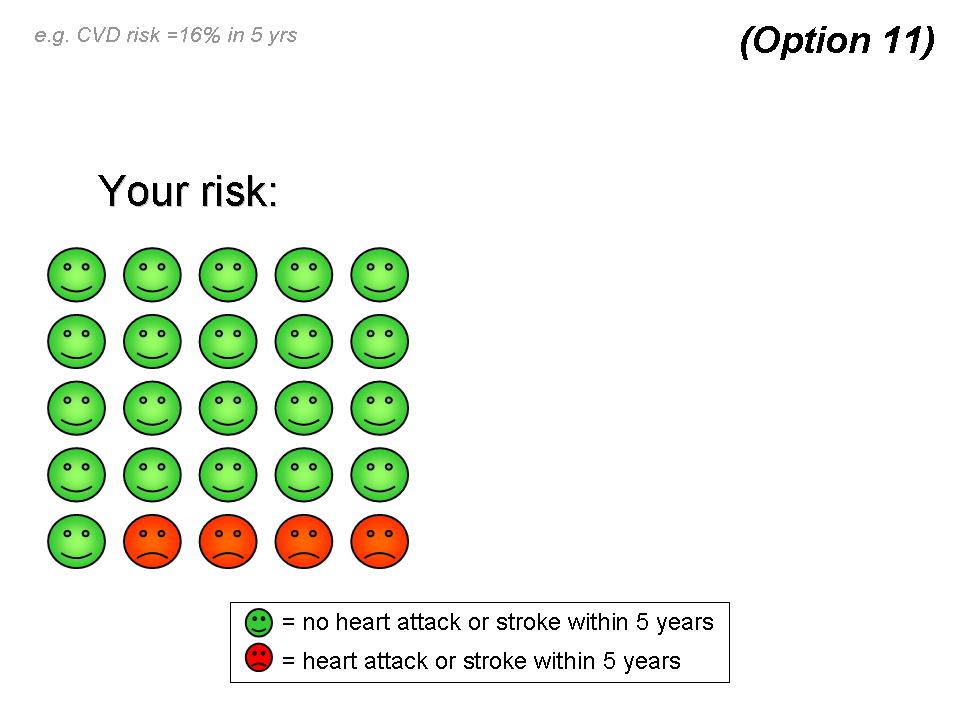


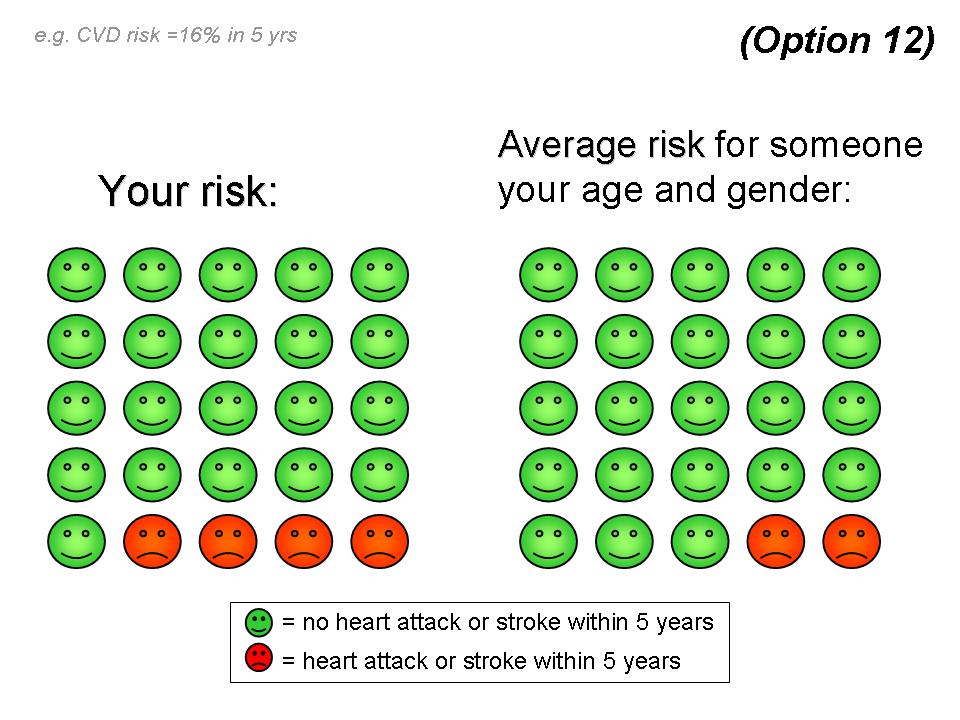


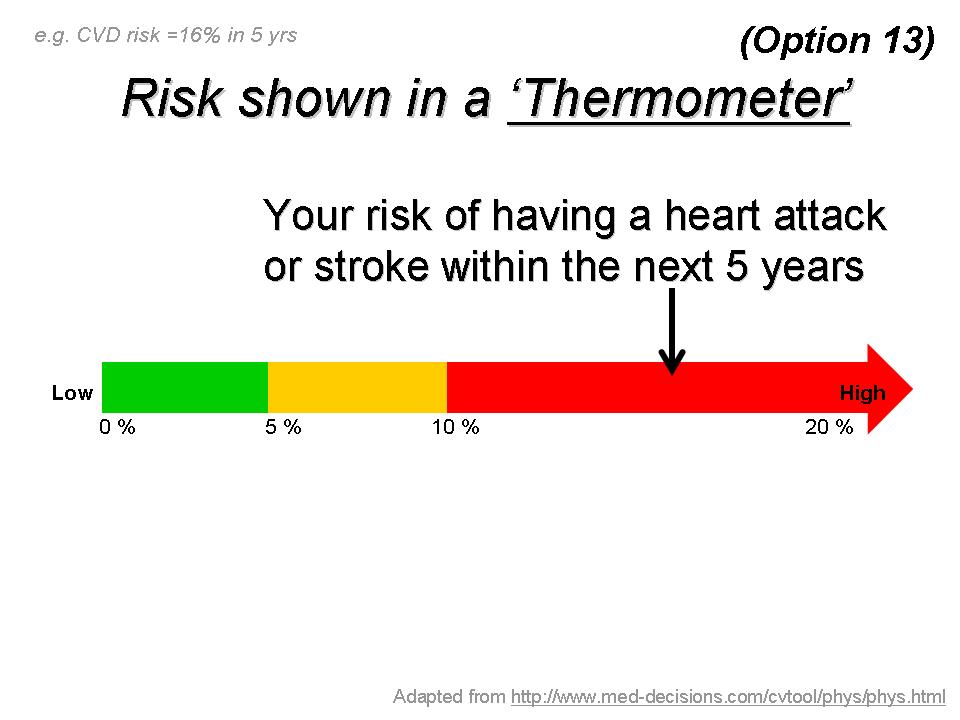


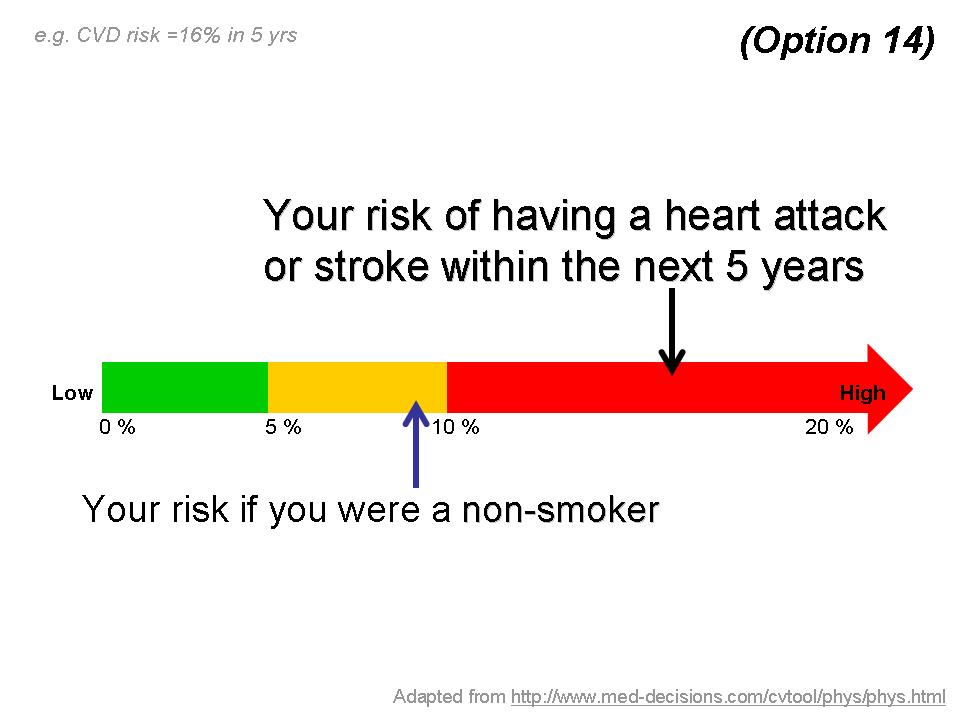


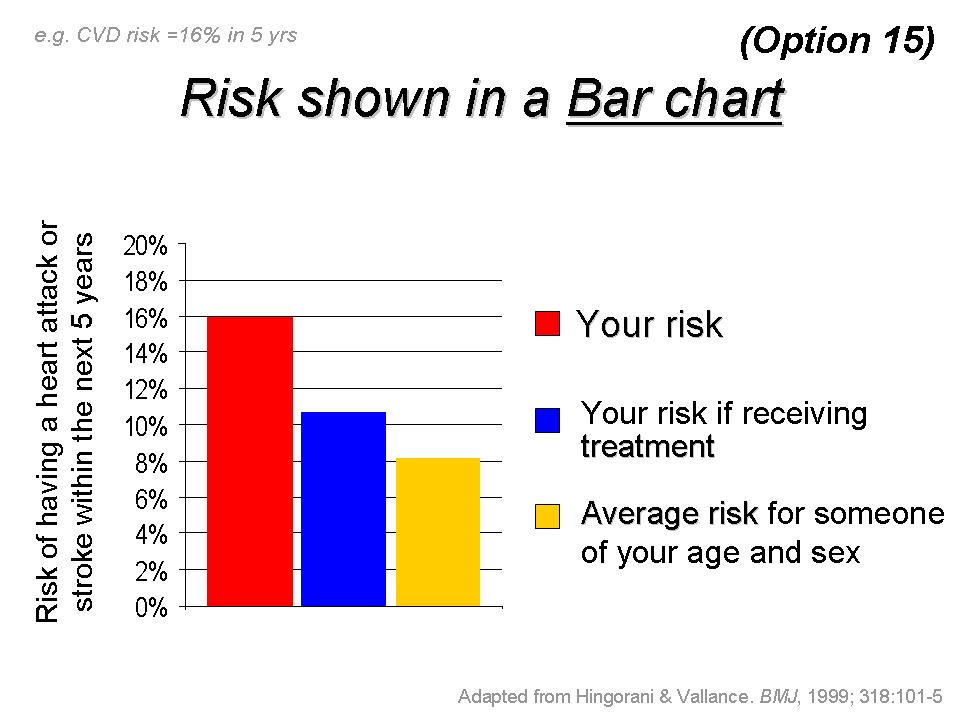


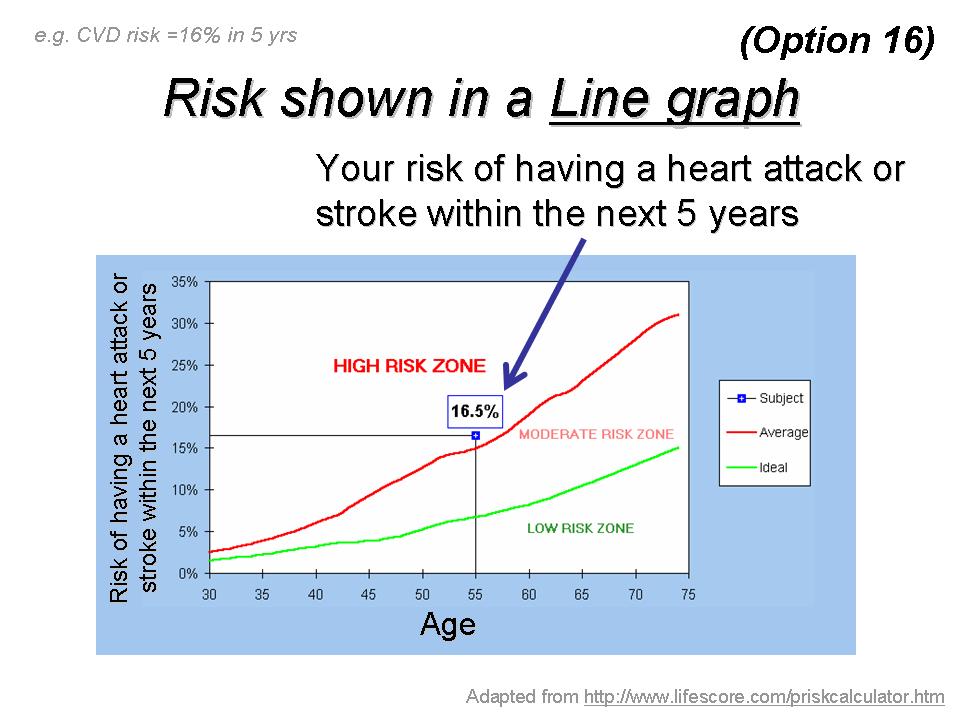


17 November 2009. Unable to trace current location of source image that was accessed in 2003 and then adapted for focus group format, and so have not been able to obtain permission to reproduce. Source web address cited.
